# Why Psychology Needs to Stop Striving for Novelty and How to Move Towards Theory-Driven Research

**DOI:** 10.3389/fpsyg.2021.609802

**Published:** 2021-01-28

**Authors:** Juliane Burghardt, Alexander Neil Bodansky

**Affiliations:** ^1^Department of Psychology and Psychodynamics, Karl Landsteiner University of Health Sciences, Krems, Austria; ^2^Department of Social Psychology, Universität Hamburg, Hamburg, Germany

**Keywords:** explorative research, theory-driven research, innovation, theory of science, replication crisis

## Abstract

Psychological science is maturing and therefore transitioning from explorative to theory-driven research. While explorative research seeks to find something “new,” theory-driven research seeks to elaborate on already known and hence predictable effects. A consequence of these differences is that the quality of explorative and theory-driven research needs to be judged by distinct criterions that optimally support their respective development. Especially, theory-driven research needs to be judged by its methodological rigor. A focus on innovativeness, which is typical for explorative research, will instead incentivize bad research practices (e.g., imprecise theorizing, ignoring previous research, parallel theories). To support the advancement of psychology, we must drop the innovation requirement for theory-driven research and instead require the strongest methods, which are marked by high internal and external validity. Precise theorizing needs to substitute novelty. Theories are advanced by requiring explicit, testable assumptions, and an explicit preference for one theory over another. These explicit and potentially wrong assumptions should not be silenced within the peer-review process, but instead be scrutinized in new publications. Importantly, these changes in scientific conduct need to be supported by senior researchers, especially, in their roles as editors, reviewers, and in the hiring process. An important obstacle to further theory-driven research is to measure scientific merit using researchers’ number of publications, which favors theoretically shallow and imprecise writing. Additionally, it makes publications the central target of scientific misconduct even though they are the main source of information for the scientific community and the public. To advance the field, researchers should be judged by their contribution to the scientific community (e.g., exchange with and support of colleagues, and mentoring). Another step to advance psychology is to clearly differentiate between measurement model and theory, and not to overgeneralize based on few stimuli, incidences, or studies. We will use ideas from the theory of science to underline the changes necessary within the field of psychology to overcome this existential replication crisis.

Many factors have contributed to the current replication crisis (see Kerr, 1998; Ioannidis, 2005; Simmons et al., 2011; Casadevall and Fang, 2012; Giner-Sorolla, 2012), which exposed the low replicability of effects in psychological science (Open Science Collaboration, 2015). Replicability is directly tied to the accuracy of measurements (Stanley and Spence, 2014), which is intertwined with strong theorizing. Both are objectively weaker in psychology than in other disciplines (see Fanelli, 2010). Although frustrating for researchers this, in and of itself, does not imply that psychology is failing. Instead it reflects the age of psychology as a comparatively young discipline in the scientific cannon—while researchers have been studying physics for over 400 years (counting from Galileo Galilei or William Gilbert) experimental psychology has only had 150–180 years to develop (counting from Gustav Fechner or William James). In addition, psychology explores a highly complex research object. Humans are compiled of those very objects physics is studying (i.e., particles or strings); plus, its interactions. It is therefore unsurprising that psychological theories do not *yet* provide strong predictions and constraints (Fanelli, 2010). However, other factors grounded in sociocultural processes do hinder the advancement of psychology. If unchecked they will prevent psychology from becoming as precise as physics. Drawing on the philosophy of science and its insights we will outline five current challenges to psychological research and possible solutions to advance the maturation of psychological science from explorative to theory-driven research.

## Challenge One: How to Promote Researchers That Advance the Field

The philosophy of science has struggled with the so called “demarcation problem,” which defines science and distinguishes it from other human endeavors. This struggle illustrates the difficulty to define the quality of scientific research. To solve this problem, Fleck (1980) argued that research is defined by its fellowship. He described science as a set of social actions, which lead to the development of a collective thought style. A collective thought style implies a certain view of the world (e.g., determining which questions are scientific and worth answering). It defines its own language and appropriate methods to investigate the world in order to gain answers to scientific questions. In an extreme reading of his thoughts, science is nothing more than a social construct. For him, the “truthiness” of scientific facts rests within the breadth and depth of the fellowship of them. Fleck’s (1980) constructivist approach to science highlights a specific problem still prevailing in current psychology: Scientific merit cannot be objectively inferred from a theory or a finding. It is thus, very difficult to evaluate the scientific merit of researchers in an impartial and unbiased way.

A socially accepted workaround has become to use the number of publications as a criterion to judge scientific achievement and to evaluate individual researchers. While it is unclear how much faculties and funders actually rely on this criterion to make hiring, promotion, and funding decisions, it is clear that it has become proverbial to publish or perish in order to succeed in science. As a result, researchers strive to increase their publication output.

A presumed advantage of the “number of publications” criterion is its perceived objectivity. However, this objectivity is spurious. Among others, authorship is influenced by social processes and not truly based on the amount or scientific merit of contributions to a publication. For instance, it is arbitrary who receives a co-authorship. Senior scientists may contribute less than junior scientists; sometimes being chief of the department and proofreading the manuscript can suffice to receive a co-authorship as a senior researcher (Stroebe et al., 2012, n. 7). Further, some evidence suggests that gender influences publication outcomes. One study found that female Ph.D. students are less likely to author papers than male Ph.D. students, even though they put in more time (Feldon et al., 2017). Papers with female first authors are reviewed longer and more critically than those of male first authors arguably because they are held to higher standards (Hengel, 2017; Fox and Paine, 2019), which would make it more time-consuming for women to publish.

Importantly, the number of publications does not consider the quality of articles. Number of publications rewards publishing many, potentially theoretically shallow articles. As an unwanted consequence psychological literature is inflated by many “parallel” theories (Glöckner and Betsch, 2011), a plethora of “sexy” singular effects (Fiedler, 2017), different “mini-theories” (Glöckner and Betsch, 2011), or simple analogies, which suffer from low degrees of precision and universality^1^. The lack of precision in theory building frequently eliminated the possibility to test theories against each other (Glöckner and Betsch, 2011) because they did not contain enough assumptions, thus, allowing multiple theories to coexist. Psychological effects were often reported outside of established theoretical structures, thus, ignoring existing theories and undermining the integration of knowledge into an overarching theoretical understanding. Variables highly similar to previously existing constructs were often introduced without referring to the related construct (“déjà-variable”; Hagger, 2014). We believe that the reason for this phenomenon, is often not a lack of knowledge, but the understanding that avoiding controversial claims by producing theoretically vague writing will increase the publication chances and facilitate the review process because it invites less scrutiny by critical reviews. To increase output and withstand the pressure to “publish or perish” highly similar articles were produced or series of studies were divided into multiple articles (i.e., salami publishing).

Thus, using number of publications as a measure for scientific merit is problematic, in and of itself. In addition, it produces high long-term costs for the whole field. The inflated literature, produced to be hired, increases the amount scientists need to read to identify new information. Furthermore, scientists spend more time reviewing articles of other researchers (Casadevall and Fang, 2012). Additionally, the need to publish for one’s own career leads to a rat race, which does not reward rigorous and hence time-consuming research. This rat race is won by fast and effective publishing and foreseeing reviewers’ reactions to one’s own submissions. Hence, authors write to please and convince the gatekeepers within the publication system. This discourages from reporting ambiguities within research, thereby leading to tactical omissions and hence, toward the behavior underlying the replication crisis. Further, the focus on publications for evaluating individuals will make these publications the main target of scientific misconduct and other manipulations, even though these publications are the main source of scientific knowledge for the general public.

Thus, incentives within psychology have created strong conflicts between behavior that advances science and behavior that advances the career of individual researchers (Casadevall and Fang, 2012; Giner-Sorolla, 2012; Nosek et al., 2012; Pickett, 2017). An optimal scientific environment needs to create as much overlap between what is good for science and the scientist as possible (see Pickett, 2017). To reduce this conflict, promotion and hiring criterions need to be based on behavior that supports the integrity of science in the long run. As a first step, publications need to become more valid measures of scientific merit. Thus, contributions of individual authors need to be identifiable (Casadevall and Fang, 2012). Of course, this can only be a makeshift solution. We are convinced that number of publications should to be dropped as a hiring or evaluation criterion in favor of in-depth evaluations of the scientific quality of publications (Casadevall and Fang, 2012). Some institutions have already adjusted their procedures accordingly and invite candidates to submit a limited list of articles. For instance, the German Research Foundation limits the number of publications per applicant to ten (Deutsche Forschungsgemeinschaft, 2020). Importantly, if institutions and senior researchers choose to favor quality of research over quantity, this must be effectively and repeatedly communicated to create a norm within the field. We recommend faculties that support a quality criterion to commit to this in job postings. Even faculties that never relied on the number of publications should make this public to support the quality orientation within the field.

Instead of focusing on publications, Pickett (2017) suggested promoting researchers that have positively impacted the scientific system. Excellent researchers increase the influx of talented new researchers, support the productivity of other scientists, and make the field a better and more productive place. This stands in contrast to researchers who hoard resources, practice favoritism, or generally conduct their projects in a competitive rather than a collaborative manner (see Anderson et al., 2008). Researchers with positive impact on the field should regularly assist colleagues by sharing ideas, materials, programs, and advice. Further, they should have fostered mentees, especially, diverse mentees are a strong indicator of excellent collaboration and leadership skills. To measure this, it is possible to use websites like google scholar or ResearchGate, which offer lists of frequent co-authors. Lists with diverse co-author’s (e.g., regarding gender, nationality, or ethnicity) are a sign of someone with a history of working with people form many backgrounds. Promoting researchers who support scientists from diverse backgrounds is not simply an idle moral goal; diversity is crucial for knowledge accumulation. Excluding input from researchers from specific groups (e.g., first generation academics, women) results in a tremendous loss of know-how and expertise for science (Merton, 1979). As science is quintessentially a collaborative endeavor, individuals that rely solely on the expertise of a small group of socially homogenous fellow researchers will inevitably constrain the potential of the field. There is ample evidence for the increased productivity of diverse teams in experimental and field studies in a wide variety of tasks (Roberson et al., 2013). Importantly, the behavior toward colleagues and mentees from minority or minoritized groups is a potent indicator of problematic interpersonal behavior (i.e., bullying or discrimination). For instance, bullies prefer victims with less power, such as subordinates and members of disadvantaged groups (Salin, 2003). Thus, having mentored individuals with diverse background is a litmus test of an excellent leader, which is what a principal investigator should be. As a means to this end, some universities let undergraduate students evaluate job candidates for faculty positions. Undergraduates are more likely to trigger problematic interpersonal behavior. Requiring principle investigators to have a history of supporting others, especially subordinates, benefits both the scientific productivity as well as the working atmosphere. Thus, scientific merit should be evaluated by criterions that have a long-lasting positive effect on the academic system and not by criterions, which lead to negative consequences. Fleck (1980) conceptualized science as a social endeavor. It should be as inclusive as possible and not a race of individuals to be the single most prolific publisher.

## Challenge Two: Defining Which Research Should Be Published or Funded—The Age of Discovery Is Over, Now It’s Time for Puzzle-Solving

Even though our recommendations suggest to devalue the number of publication criterion, publishing one’s findings is essential to science. The criteria used to evaluate manuscripts within the peer-review process are a crucial factor that shape the scientific conduct. We will draw on Kuhn’s (1970, 1996) insights into the theory of science to scrutinize the review criteria. Kuhn describes that science evolves through a progression of different phases. Early stage science, termed protoscience, develops into paradigmatic science^2^. Protoscience (still) lacks broad theories and thus, often relies on explorative as opposed to theory-driven research. It therefore explores ideas in a relatively random fashion and uses idiosyncratic methods. We argue that psychology is in the process of developing from protoscience into paradigmatic science. For this transition, the field has to adopt and consent to a paradigm. A research paradigm defines relevant theories, (measurement) instruments, values and metaphysical assumptions, which are kept relatively constant. To succeed, each research community needs to commit to a paradigm, to meaningfully communicate with each other and to explain as many findings as possible in a way that can be shared, taught, and utilized. Explorative research has to cease when the scientific community consents on a paradigm.

Kuhn (1996) described research conducted within paradigmatic science as “solving puzzles,” meaning, that researchers have a rather clear understanding of what they expect to find prior to conducting their studies and of the tools needed to show the predicted effects. They need to fill the gap with the missing piece; the solution of the overall puzzle is well-defined. A research finding may lead to changes in the details of the auxiliary assumptions or measurement models (see below). However, the paradigm as well as the associated theory should remain unchanged.

Only when a critical number of anomalies and unexplained findings accumulate will researchers begin to question their paradigm. In the subsequent “revolutionary phase” a new scientific paradigm is developed. These phases of revolution, however, are rare and do not constitute everyday research practice. Thus, revolutionary science is unusual and more importantly cannot be planned ahead, for instance, within tenure-track or a grant proposal. Hence, scientists should not strive for it. Instead, they should strive to conduct research in the spirit of a Kuhnian paradigmatic phase, which is marked by high precision and rigorous application of sound methods—not by breakthroughs. This research emphasizes “dull” routine jobs like theory improvement or increasing the precision of measurements as the essence of good (paradigmatic) research.

As a consequence, new criteria for evaluating submissions or grant proposals that promote good paradigmatic research need to be established. If we follow Kuhn’s logic and we believe that psychology needs to transition into a paradigmatic science, then “innovativeness” can no longer be used as an adequate criterion to judge research, because it counteracts the objectives of paradigmatic research. Currently, “innovativeness” is frequently used as a central criterion to evaluate the contribution of an article or research project (Giner-Sorolla, 2012; Nosek et al., 2012). Innovation is equated with “novelty,” implying that an unknown effect or a new theory is preferred. Thereby, the criterion of innovation actively disincentives paradigmatic science: Studies not seen as novel enough can easily be dismissed as “trivial.” Results are frequently rejected by journals because they are “merely incremental” (Giner-Sorolla, 2012; Organization Science, 2020), which creates incentives that hinder good paradigmatic research. For instance, repeating a study on stereotypes with the gender category instead of the race category is an ideal study in paradigmatic science, because it tests whether the existing assumptions hold for new stimuli. Testing whether previous assumptions hold for other stimuli is furthermore also a test about the ceteris paribus conditions (when all other things are equal) of a theory; meaning that results replicate. It is thus, good paradigmatic research. These “dull” research programs truly add to the accumulation of robust knowledge instead of piling up fancy, new looking fast fashion research.

Furthermore, the focus on innovation has the tendency to undervalue research that draws attention to old but ongoing problems for which a solution has already been suggested (e.g., Clark, 1973; Judd et al., 2012). The community should not assume that a point is made successfully just because someone already made it. Currently such valuable “reminders” are either unpublishable or relegated to less prestigious outlets. This tendency understates their scientific contribution and prevents arguments from informing future research. The emphasis on novelty remains unchallenged even though recent replication attempts have highlighted the sophistication involved in a successful replication of a previous study (e.g., Maxwell et al., 2015; Noah et al., 2018; Bressan, 2019).

To overcome these problems, we advocate to redefine the value of scientific contributions, especially, to stop valuing innovation and novelty. Following Kuhn’s observations about paradigmatic science, unpredicted results are more often than not a sign of imprecise theories. They are thus, not something researchers should strive for. As shown above, rewarding innovation in science has the tendency to hinder incremental work. Researchers should systematically test predictions made by their theory. In contrast, a focus on innovativeness will have multiple negative effect: First, innovativeness is often much more difficult to proof than accuracy. Accuracy can, at least partly, be quantified in terms of reliability and validity; thus, offering relatively objective measures. Therefore, judging the accuracy of a study should show high interrater (i.e., reviewer) consensus (e.g., a study may explain 10% more variance in a given design than previous studies). However, innovativeness is much more difficult to recognize let alone to quantify. Innovativeness is often identified by an absence of a similar study, theory, or effect. This absence is, however, only recognized by a reviewer that has a perfectly fitting expertise. Something that is currently difficult to ensure but will become increasingly difficult due to the increased differentiation within the field. Thus, the nature of the innovativeness criterion makes it much easier to cheat by omitting relevant work and much harder for reviewers to recognize. How incapable we are to identify good work when seeking innovation becomes clear from the fact that work that later Nobel prizes were based on, has consistently been published in less prestigious journals (Kumar, 2009).

Second, a logical outcome of preferring novelty over accuracy is that methodologically more advanced follow-up studies are frequently published in lower ranking journals than methodologically less advanced and less accurate first demonstrations (e.g., Sherman and Bessenoff, 1999; Sherman et al., 2003), which undermines their value. The easiest remedy for this is to use an ongoing review system for articles (Nosek and Bar-Anan, 2012). This would imply that, similar to online rating engines, publications could constantly be (re)rated according to their methodological rigor and impact on the field. This would assure that over time the methodologically more rigorous study would have a better rating than the earlier, less precise versions and thereby, would receive more attention from researchers. This solution is technically easy to implement. A more comprehensive solution would imply to abandon the classical journal system and instead publish all articles in the same database. Journals would merely “advertise” articles, which is already successfully implemented in the field of physics (Nosek and Bar-Anan, 2012).

Third, the negative effects of focusing on innovativeness can even reach beyond the academic system when researchers decide to disseminate early findings without replications. For instance, the effect presented in the most watched TED-talk (i.e., power posing) is now challenged (Jonas et al., 2017). This is unsurprising since the corresponding studies were published only 2 years prior to the talk (Carney et al., 2010). Such fast communication of unreplicated results bears the risk to delegitimize science and its conduct in the eye of the general public.

Fourth, the difficulty to measure innovativeness introduces additional ambiguity into the review process. Ambiguity is known to increase the impact of stereotypes (e.g., Norton et al., 2004) and as a consequence racism, sexism, ageism and other biases such as motivated reasoning steaming from conflict of interests. Since innovativeness is associated with scientific merit it is part of the ideal that individuals need to fulfill to become researchers. Such norms will likely influence hiring and career decisions via other- and self-selection (Eagly and Karau, 2002; Heilman, 2012). It is thus noteworthy, that women are less likely to describe their research as “new” or “innovative” than men (Lerchenmueller et al., 2019). Therefore, the innovativeness criterion may elevate barriers women perceive when entering or pursuing a career in science.

Fifth, a caveat of innovation is its association to creativity. Innovation and creativity are linked to dishonesty (Khessina et al., 2018). Both, a creative disposition and a creative mindset were shown to increase cheating behavior (Gino and Ariely, 2012; Gino and Wiltermuth, 2014). This is presumably because seeking creativity encourages actors to ignore rules and thereby provides a justification for unethical behavior. Giner-Sorolla (2012) argued that creativity is linked to an artistic conceptualization of science in which output has to be aesthetically pleasing and therefore narratives and data have to be “beautiful.” This contradicts the reality of empirical research and therefore potentially motivates problematic conduct.

Sixth, relying on accuracy instead of innovation might even reduce the negative effects of “scooping.” Scooping, in its most extreme form, implies that a researcher publishes a study that they copied from another researcher, for instance from a conference presentation. As a result, the person who originally had the idea will find it difficult to publish it. If being the first was valued less, it should become more rewarding to advance ideas than to simply copy them. As a result, both studies would be publishable. It might even become more beneficial for both researchers to collaborate than to compete.

Thus, the development into a paradigmatic science makes innovativeness an obsolete criterion to judge publications, grants, and job candidates on. Psychological science should instead emphasize the value of accuracy. Accuracy by its nature, needs to be evaluated continuously. Even though Kuhn (1970) explicitly refrained from providing advice about how to arrive at the paradigmatic stage, it is clear that it requires both broad and precise theories. Agreeing upon such an overarching theory must be a crucial goal in psychology. Some broad models already exist; however, they are not widely adopted. A successful model needs to integrate many different assumptions for instance, about psychological constructs and about the brain’s architecture. The Hierarchically Mechanistic Mind (HMM) theory offers such a broad framework^3^. Grounded in evolutionary psychology, it combines assumptions from psychology and neuroscience to outline perception, cognition, and behavior based on a hierarchical, dynamic brain architecture. Such a highly universal model, would need to be integrated with models with higher precision in specific areas. For instance, the PSI theory (Kazén and Quirin, 2018), which integrates assumptions about motivational and volitional processes to explain personality. In the resulting universal and precise framework, future researchers would fill in well-defined gaps in knowledge with the matching “puzzle piece.”

## Challenge Three: How to Conduct Good Research

If Kuhnian “puzzles” ought to be solved, then there must be a scientific methodology to solve them. Popper argued that the scientific process implies that researchers choose a theory, deduce hypothesis from it and test them empirically. If the data doesn’t support the hypothesis, the theory should be given up. This idea was revised and refined by Lakatos (1970), who argued that in order to derive a research hypothesis from a theory, researchers need to make additional auxiliary hypotheses and assumptions. Further, specific measurement methods and theorizing about these measures (measurement models) are needed to gather data. Finally, all hypothesis can only be tested under ceteris paribus conditions (when all other things are equal, and no intervening factors present). Research following Lakatos has to reconcile all these components to test the proposed hypothesis. If the empirical data does not fit the stated research hypothesis, it is not possible to identify the source of this imbalance due to the many factors involved in the process. The reason for the data not matching the hypothesis could lie within the theory, the auxiliary hypothesis and assumption, the stated hypothesis, the measurement, the theory of measurement, or not fulfilling the ceteris paribus conditions. The same can also be true, if the data *does* match the proposed research hypothesis. Since, this could also result form an error within the research process; for example, a confounded measurement. Therefore, data alone can neither verify nor falsify a theory. Rather, data can initiate a reconsideration of the whole research process and its parts. This requires fine tuning and adaptations in every part of the research process and repeated tests of scientific assumptions against new data. Thus, scientists have to balance all elements of the research process (theory, auxiliary hypothesis, hypothesis, measurement, ceteris paribus and data), while not knowing, which of these elements is ultimately false, and which can be relied on until it also has been proven wrong. Lakatos (1970) compared the research process to drawing piles into a swamp to erect a building on top of them, not knowing which pile would last for the time being.

Lakatos’ (1970) conceptualization of science helps to outline ideal theory-driven research. The implications especially pertain to the publication process. Since the replication crisis uncovered many gaps in our knowledge, some scientists may respond by displaying more restrain in postulating assumptions and therefore make fewer predictions than previously. In contrast, Popper and Lakatos urge individual researchers to make clear predictions and claims. The goal they propose is not to be proven correct—quite the contrary, theories are by default incorrect because they oversimplify—instead, making clear predictions and be proven wrong is the ideal way to advance theory development and hence understanding. This misconception frequently surfaces, when theories are tested against each other by researchers who claim to be impartial about their preferred theory. This impartiality can be motivated by trying to sell a null effect finding or to assure *a priori* that any outcome of an experiment could be valid, guaranteeing a publication. According to Lakatos (1970), however, testing theories against each other necessitates the opposite of impartiality. Researches ought to take a stand on which theory is better and why. They need to show a “dare-devil attitude” (Lakatos, 1970, p. 112), not an impartial one and hence should be motived to prove that their theory is better. In extreme cases of impartiality, researches “pick” different theories for each publication based on their results, while never accumulating the information they found into a stance that could be proven wrong. They thereby circumvent the burden of accountability. Accumulating restraining assumptions is crucial for theory development and hence, necessary for scientific progress. Establishing boundaries of one’s own theory should be a success and not a stain. To overcome these obstacles we reaffirm the suggestions made by Glöckner and Betsch (2011): Scientists need to (1) make strong claims (“state a finite set of definitions and propositions that together constitute their theory” p. 717), (2) “formulate the propositions in such a way that the theory as a whole clearly predicts particular states of the world to occur and others not to occur.” (p. 717), and (3) “The authors should be obliged to explain which kind of empirical observations they would consider a fundamental violation of their theory” (p. 717).

The reluctance to take a strong stand about theories and predictions seems to be based on the expectation that these preferences are often attacked within the review process. This is a crucial obstacle for psychology’s transition into a paradigmatic science. Theoretical assumptions and individual preferences should not be buried in the review process. In a beneficial review process reviewers and editors have to assure the logical clarity of the theory, hypothesis, auxiliary assumptions, measurement, and ceteris paribus conditions. However, personal preferences for one theory or hypothesis over another should never influence the course or outcome of a review. Any in principle reasonable (i.e., logically consistent and empirically not repeatedly disproven) assumption is a valid contribution to a theory, irrespective of one’s own preferences. The review process is not the right outlet to criticize assumptions that seem at odds with reviewers’ or editor’s own views. Reviewers and editors should relegate such criticism to a new publication or comment.

However, for the field to change successfully, it is not sufficient to act in line with these suggestions. Importantly, senior researchers should explicitly commit to them by welcoming reasonable hypotheses, as well as supporting explicit assumptions and constrains within the review process (maybe sport a “theory support” batch). No author will follow these guidelines until a critical mass of senior researchers (i.e., editors, reviewers, and supervisors) explicitly supports them.

The main task of reviewers and editors should be to assure the logical clarity of the theory, hypothesis, auxiliary hypothesis and assumptions, measurement, and ceteris paribus conditions. Contradictions between assumptions within a submission need to be resolved. Predictions that conflict with previous findings need to be discussed. They should only be changed if there is an overwhelming number of evidences against them. In this case the article should argue about moderating conditions or context influences that explain why authors assume that a prediction that ostensibly contradicts previous findings is valid. Importantly, research should not be solely judged by its findings, rather the research process as a whole should be evaluated. As Lakatos (1970) pointed out, science is not about findings or data but the interplay of data with theories, auxiliary assumptions, measurement methods, and ceteris paribus. Currently, many reviews put too little emphasis on the measurement models and their adequacy. To evaluate the appropriateness of the review process and to prevent biases, the entire review process should be published alongside the article to allow other scientists to evaluate the process (Nosek and Bar-Anan, 2012; Wicherts et al., 2012).

Lakatos’ (1970) ideas define good research as theory-driven. However, based on psychology’s short history, it occasionally cannot provide a theory or lacks a sufficiently precise estimate of an effect. Without a scientific paradigm, a young discipline needs to establish effects in an explorative or descriptive manner. It is not useful to force authors to retroactively apply a theory to their findings if the study was not conducted based on said theory or was data-driven. The quality of this descriptive research should be judged based on its methodological rigor (great recent examples provide Smith and Hofmann, 2016; Zwebner et al., 2017; Ray et al., 2019). Better descriptive research has higher internal and external validity. It should create assessments closer to reality, use more accurate dependent measures, bigger and more representative stimulus and participant samples, or non-reactive measures. However, once a theory is established it should be used until it is refuted, or a better theory is proposed. Better theories are marked by higher precision or universality (Popper, 2002). They include more constrains and hence, are more likely to be falsified. Additionally, a new theory that repeats old predictions and constrains but puts them into a broader context is an important improvement because it increases universality. Small theories or singular findings without theoretical foundations should increasingly disappear even, if they are “sexy” or surprising (Fiedler, 2017).

Even though, theory-driven research must be the goal, it is in general, unproblematic to engage in explorative research. The main risk for science steams from mixing theory-driven with explorative approaches. A highly popular chapter on academic writing illustrated such a mix (Bem, 2002). The chapter encouraged researchers to analyze data in any way possible—which is adequate in explorative research—however it also advised scientists to write the results as if they had been predicted, irrespective of the original predictions. Thus, the explorative analysis was followed by theory-driven elaborations; explorative and theory-driven research were inadequately mixed. Where explorative research is sold as theory-driven, the data is used twice: First, to discover a new hypothesis and second, to test that hypothesis (Kerr, 1998; Wagenmakers et al., 2011). Thus, researchers skip the necessary replication. Data is no longer used to challenge the theory, auxiliary assumptions, or measurements, instead the theory is picked in response to the data.

In conclusion, scientists in psychology currently conduct two different types of research: theory-driven and explorative research. Until psychology adopts overarching paradigms, it seems useful to preserve both types of research since explorative research can be fast to provide new impulses. They should, however, be clearly separated from theory-driven research. The most straightforward solution are separate journal sections reserved for explorative/descriptive and theory-driven research. These sections should apply different evaluation criteria and approaches. Contributions for theory-driven sections would be judged by their increase in accuracy in theory and measurement (see also Wagenmakers et al., 2011), while explorative findings could be judged by their internal and external validity. The differentiation into sections would support the implementation of different evaluation criteria and create a demand for theory-driven research. Thus, it would incentivize researchers to conduct theory-driven research and also elevate the value of theory-driven research. The long-term goal for psychology would remain to transition into theory-based research.

## Challenge Four: Harmonizing Theory and Evidence

Holzkamp (1981) distinguished between theoretical sentences and empirical sentences: Theoretical sentences are generalized ideas (e.g., stereotypes influence behavior), while empirical sentences are statements about specific observations (e.g., Jamal is described as more threatening than David). A subtype of empirical sentences are experimental sentences that pertain to specific observations within experimental settings in contrast to naturalistic settings. Holzkamp (1981) argued that experimental sentences can be understood as “now-and-here-data” meaning, that they are the result of the given experimental context (e.g., room, demand effects, stimuli, experimental method, experimenter). His work emphasized the gap between experimental sentences and empirical sentences: Experiments are artificial products and can only try to approximate the empirical reality outside of the laboratory. The gap between experimental sentences and empirical sentences is rather large within the field of psychology as the laboratory does not resemble everyday life. Instead, it is a highly artificial environment in which people act as test subjects. This limits their ability to act as independent agents, which renders their behavior irrelevant to everyday life. This gap is problematic, but theoretically it could be bridged by clever experimental designs. There is however a second gap, between empirical and theoretical sentences, which is even more relevant. Theoretical sentences always have multitudes of possible meanings, while each empirical sentence can only represent one of these meanings. But, if there are multiple meanings to each theoretical sentence, then any empirical sentence is never equivalent with the complete meaning of any theoretical sentence. Holzkamp (1981) concluded that no “here-and-now-data” can ever verify or even falsify theoretical sentences. Thus, measurements should never be equated to assumed theoretical constructs.

This insight is crucial for the advancement of psychology: The benefits of theory-driven research might tempt some researches to infuse all levels of analysis with theoretical meaning. However, theories need to be strictly separated from measurement models and thus concrete observations. For instance, behavioral observations need to be separated from the mental processes that are assumed to cause them (see De Houwer et al., 2013). Measurement methods need to be separated from the presumed underlying theoretical constructs and processes (Sherman and Klein, in press). It would be wrong to jump to the conclusion that using an “implicit measure” amounts to having measured only “automatic” processes (e.g., Calanchini et al., 2014). Put differently, psychological research should refrain from over-theorizing empirical data; that is, equating theory and measurement model.

Holzkamp’s (1981) distinctions highlight the necessity to bridge the gaps between empirical and theoretical sentences in the most consistent, reliable, and transparent way. This highlights the crucial need for replications in different contexts, under different circumstances, with different stimuli and participants. These replications generate a plethora of empirical sentences that potentially are instances of theoretical sentences. However, for this to succeed, theories need to define as many auxiliary conditions for an effect to occur as possible, as this will increase the chance fur successful replication. For instance, Noah et al. (2018) suggested that a previous high-profile replication effort of the facial-feedback effect had failed because it introduced video recordings to the original paradigm. The replication seemed to have failed because the feeling of being observed blocked the predicted facial-feedback effect. Thus, introducing this assumption into the theory will increase replication chances. Well defined theories, with more specifications about context and moderators will allow a more thorough evaluation of the success or failure of a replication. For instance, Bem (2011) suggested that people can respond to stimuli that will be presented to them in the future (precognition). Within his set of studies, the effect was found for erotic stimuli and sometimes for neutral stimuli, but sometimes it was absent for neutral stimuli. The effect on neutral stimuli could be interpreted as an unsuccessful replication. However, no rational was provided as to when valence should or should not affect precognition (Bem, 2011; Rouder and Morey, 2011). Therefore, Bem (2011) argued that the replication was successful, while Rouder and Morey (2011) argued that it was unsuccessful. These conflicting interpretations highlight the necessity to clarify circumstances, moderators and ceteris paribus for every theory as extensively as possible, so that replications and their success are less open for interpretation.

## Challenge Five: Establishing a Checklist for Good Research

Latour and Woolgar (1986) described how statements develop into consented upon scientific facts. According to their analysis, science is based on “literary inscriptions.” This means that scientists use numbers and words as placeholder to study natural phenomenon by ascribing digits or labels to these natural entities. Without assigning numbers or language to researched phenomenon (e.g., coding if a participant is a man or a woman) it is impossible to systematically study anything and derive conclusions about recurring trends. Therefore, literary inscription is the first step to construct order in an, in other ways, chaotic system. Latour and Woolgar (1986) pointed out that scientific facts are the product of “sorting, picking up and enclosing” (1986, p. 247) of the inscriptions given to the studied material. Sorting entails which studied material is inscripted and how. Picking up entails the conscious decision to look at certain inscriptions (e.g., man and women) and not at others (e.g., non-binary individuals) and enclosing entails the act of integrating inscriptions in a way that an effect or scientific fact can be seen, for instance, within a statistical diagram.

“The whole series of transformations, between the rats from which samples are initially extracted and the curve which finally appears in publication, involves an enormous quantity of sophisticated apparatus (…). By contrast with the expense and bulk of this apparatus, the end product is no more than a curve, a diagram, or a table of figures written on a frail sheet of paper. It is this document however, which is scrutinized by participants for its “significance” and which is used as “evidence” in part of an argument or in an article” (Latour and Woolgar, 1986, p. 50).

The sophisticated apparatus, which is necessary to sort random noise from relevant data and to gain agreed on facts is cost intensive. From Latour’s and Woolgar’s perspective scientific facts are not so much found but manmade things. They are constructed under rules. Science is seen as the human striving to organize the world by choosing to look at “what” and “under which conditions” in order to mold an entity into a reliable, agreed on phenomenon. The only thing hindering the production of infinite scientific facts are the costs associated with the production of facts. Therefore, science is seen as an ongoing project that seeks the most cost-effective way to organize the world. This view on science highlights the importance of the rules that guide the process to establish agreed upon scientific facts and leads us to propose 10 procedures, which should be adhered to.

First, the methods of data analysis should be defined prior to the analysis (Simmons et al., 2011; Wagenmakers et al., 2011). Preregistrations seem to be an ideal tool to force researches to take a stand. However, we don’t believe that this method has lived up to its promise yet. Scientists still need to appreciate preregistrations as an opportunity to make theoretical predictions and to protect themselves from self-serving biases (Nuzzo, 2015; Nosek et al., 2018).

Second, stimulus materials, computer code, and raw data should be publicly available (e.g., Wagenmakers et al., 2011; Giner-Sorolla, 2012) to allow for independent checks and further analysis.

Third, internal validity is increased by rigorous control of stimuli, which results from thorough pretests. Consequently, researchers need to evaluate and reduce confounds within stimuli. Subsequently, stimuli should either be representative or selected to be extreme on a criterion defined by the theory. They should also be made freely available to allow for *post hoc* in-depth analysis of the stimuli and to ensure easy replications.

Fourth, external validity should be increased by more naturalistic stimuli, which should be representative and span all possible categories (Brunswik, 1955, 1956). As a first step including many stimuli will be helpful, however, ideally they should represent the nature and distribution of stimuli in reality (Fiedler, 2000). Further, study settings should be naturalistic (Holzkamp, 1981).

Fifth, participants should represent many ages, sexes, education levels, or cultures. Especially, they should be relevant to the research question at hand (Henrich et al., 2010; Landers and Behrend, 2015). For instance, studies on hiring decisions should rely on raters with hiring experience.

Sixth, experimenter bias should be controlled equally strongly as ceiling effects and experimental mortality (i.e., selective dropout of participants), as well as any effects of one measure on the following measure.

Seventh, dependent variables should be representative and relevant. For instance, hiring decisions are categorical decisions, nevertheless, research commonly uses continuous measures (e.g., Uhlmann and Cohen, 2005).

Eighth, it is necessary to rule out reactive or interaction effects of testing where a pretest increases the scores on a posttest. This can be achieved within the Solomon four group design.

Ninth, effects should be replicable in multiple measures as not to fall for one measurement error.

Tenth, responses to the replication crisis have encouraged more rigorous statistical methods especially, requests to reduce the alpha-error (e.g., Nosek et al., 2018). The Bayesian approach constitutes a helpful alternative to frequentist *p*-values (Kruschke, 2014; Rouder, 2015; Dettweiler, 2019). This approach explicitly differentiates between theory and measurement models. The priors force researchers to state constrains, which is in line with our understanding of science as proposed above. The priors reinforce knowledge accumulation. Bayes allows to test null-hypothesis and benefits more from increased measurement accuracy than frequentist models that rely on *p*-values only.

However, any statistic will fail if it is applied to inapt theories. Even the most advanced statistics can’t safeguard against a system that rewards those scientists who were lucky “to find” something new. Therefore, as stated above, new publication guidelines are necessary to encourage sustainable research.

The previous sections have outlined many epistemological ideas about how science should be conducted. Previous thinkers have pointed out that scientific merit is socially constructed. They described that theory-driven research leads to better scientific outcomes when researchers are able to carefully balance theory, auxiliary assumptions, measurement models, ceteris paribus conditions, and data. Researchers should always be aware of the context and conditions they create within an experimental paradigm and how it impacts participants’ responses. Via successive sorting and picking of relevant information, the community can, however, identify the current consent within a field.

On the one hand, it seems these ideas are well established and rarely refuted, on the other hand, the scientific conduct in psychological science often deviates from these ideals. The reason for this is that the incentives within psychological science often do not support best practices. To fulfill the potential to become a fully realized paradigmatic science psychology will need to change the incentive structures to align the behaviors that are good for the individual scientist and the scientific community. We need to support the use of broad and comprehensive theories with a consistent emphasis on measurement models. The goal must be to adopt an overarching research paradigm, that includes as many aspects of human life as possible accompanied by measures for these factors. We need to request replications that generalize our findings to new stimuli, individuals, measurements, and contexts, overcoming the popularity of innovation. We need to use the review process as a means to strengthen our ideas and not to protect our theories or censor ideas that contradict our intuition. When psychology can offer broad theories over its entire area it will eventually see more successful replications and will grow as a field.

## Author Contributions

JB created the first draft. AB substantially rewrote it. Both revised the resulting draft and finalized it.

## Conflict of Interest

The authors declare that the research was conducted in the absence of any commercial or financial relationships that could be construed as a potential conflict of interest.

## Footnotes

1 Precision describes the number of possible implementations allowed by predictions. Universality describes the number of situations that the theory can be applied to Popper (2002).

2 Kuhn referred to paradigmatic science as “normal science.” As a trained physicist his focus lied on physics, which he equated with “normal science.” Since this terminology is biased, we instead use the term paradigmatic science.

3 We thank an anonymous reviewer for this suggestion.
